# Preclinical Activity and Pharmacokinetic/Pharmacodynamic Relationship for a Series of Novel Benzenesulfonamide Perforin Inhibitors

**DOI:** 10.1021/acsptsci.2c00009

**Published:** 2022-05-31

**Authors:** Kate H. Gartlan, Jagdish K. Jaiswal, Matthew R. Bull, Hedieh Akhlaghi, Vivien R. Sutton, Kylie A. Alexander, Karshing Chang, Geoffrey R. Hill, Christian K. Miller, Patrick D. O’Connor, Jiney Jose, Joseph A. Trapani, Susan A. Charman, Julie A. Spicer, Stephen M. F. Jamieson

**Affiliations:** † QIMR Berghofer Medical Research Institute, 300 Herston Road, Herston, Queensland 4006, Australia; ‡ Auckland Cancer Society Research Centre, Faculty of Medical and Health Sciences, The University of Auckland, Private Bag 92019, Auckland 1142, New Zealand; § Maurice Wilkins Centre for Molecular Biodiscovery, The University of Auckland, Private Bag 92019, Auckland 1142, New Zealand; ∥ Cancer Immunology Program, Peter MacCallum Cancer Centre, 305 Grattan Street, Melbourne, Victoria 3000, Australia; ⊥ Sir Peter MacCallum Department of Oncology, The University of Melbourne, Parkville, Victoria 3052, Australia; # Clinical Research Division, Fred Hutchinson Cancer Research Center, Seattle, Washington 98109, United States; ∇ Centre for Drug Candidate Optimisation, Monash Institute of Pharmaceutical Sciences, Monash University, 381 Royal Parade, Parkville, Victoria 3052, Australia; ○ Department of Pharmacology and Clinical Pharmacology, Faculty of Medical and Health Sciences, The University of Auckland, Private Bag 92019, Auckland 1142, New Zealand

**Keywords:** perforin, perforin inhibitors, pharmacokinetics, bone marrow transplantation, pharmacokinetic/pharmacodynamic relationship, plasma protein binding

## Abstract

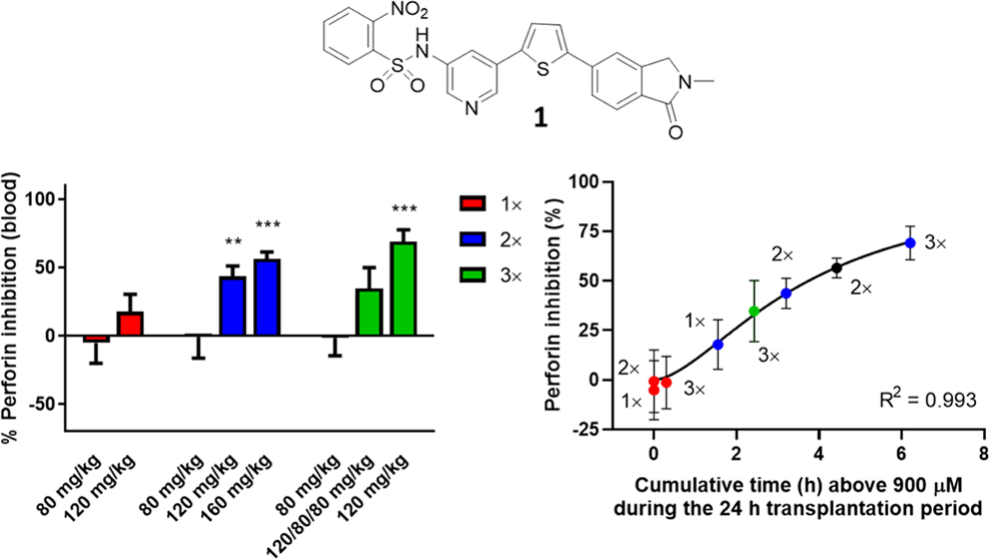

Perforin is a key effector of lymphocyte-mediated cell death pathways and contributes to transplant rejection of immunologically mismatched grafts. We have developed a novel series of benzenesulfonamide (BZS) inhibitors of perforin that can mitigate graft rejection during allogeneic bone marrow/stem cell transplantation. Eight such perforin inhibitors were tested for their murine pharmacokinetics, plasma protein binding, and their ability to block perforin-mediated lysis *in vitro* and to block the rejection of major histocompatibility complex (MHC)-mismatched mouse bone marrow cells. All compounds showed >99% binding to plasma proteins and demonstrated perforin inhibitory activity *in vitro* and *in vivo*. A lead compound, compound **1**, that showed significant increases in allogeneic bone marrow preservation was evaluated for its plasma pharmacokinetics and *in vivo* efficacy at multiple dosing regimens to establish a pharmacokinetic/pharmacodynamic (PK/PD) relationship. The strongest PK/PD correlation was observed between perforin inhibition *in vivo* and time that total plasma concentrations remained above 900 μM, which correlates to unbound concentrations similar to 3× the unbound *in vitro* IC_90_ of compound **1**. This PK/PD relationship will inform future dosing strategies of BZS perforin inhibitors to maintain concentrations above 3× the unbound IC_90_ for as long as possible to maximize efficacy and enhance progression toward clinical evaluation.

A robust immune system is critical for human health; however, it can also result in a multitude of serious pathologies when overactive or poorly regulated. The immune system’s critical cytotoxic effector cells, cytotoxic T lymphocytes (CTLs) and natural killer (NK) cells, maintain immune homeostasis by eliminating virus-infected and oncogenic cells through a process known as the granule exocytosis pathway.^1−4^ When a cytotoxic lymphocyte or NK cell makes contact with a cognate target, an immunological synapse is formed and secretory granules migrate to the effector membrane where they release their luminal contents into the synaptic cleft. This results in the plasma membrane of the target cell being exposed to the combined action of a pore-forming protein called perforin and a group of serine proteases known as granzymes.^3^ Perforin is a calcium-dependent glycoprotein that is essential for this process, forming arc- and ring-shaped transmembrane pores that cause transient osmotic disruption in the target cell membrane.^5^ This in turn enables efficient diffusion of the granzymes into the target cell cytosol where they initiate apoptosis. It is a rapid process, occurring in less than 20 s, that overwhelms the target cell membrane repair response.^6^ Remarkably, although the effector and the target cell are both exposed to perforin within the synapse, only the target cell membrane is disrupted while the CTL/NK is unaffected. This is due to the effector cell plasma membrane within the synapse being protected by a high lipid order that repels perforin, together with exposed phosphatidylserine residues that sequester and inactivate perforin. This enables cytotoxic effector cells to kill multiple target cells in rapid succession without succumbing themselves.^7^

Mutations causing complete loss of perforin expression or function can result in fatal immune dysregulation, while partial loss has been associated with a predisposition to benign immunoproliferation or to malignancies such as lymphoma and leukemia.^3^ Nonetheless, individuals who inherit two copies of a common hypomorphic perforin allele (A91V) that confers >60% reduction in *in vitro* perforin activity were shown in a recent large population study to maintain good health and to survive to the age of 75 years as frequently as those who inherit the wild-type allele.^8^ This strongly suggests that temporarily and partially reducing perforin activity by pharmacological means is likely to be well tolerated. Conversely, inappropriate or excessive perforin activity has been implicated in numerous pathologies, including insulin-dependent diabetes, postviral myocarditis, and fulminant viral hepatitis (FVH)^9,10^ as well as therapy-induced conditions such as allograft rejection and graft versus host disease (GvHD).^2,11^ These examples represent a clear unmet need for new targeted treatments of immune-related pathologies, especially as less-focused conventional therapies (such as corticosteroids) inevitably suppress much of the immune system, including many immune functions that are not relevant to the pathology. Because perforin is indispensable for the entire cell death pathway’s function, it has significant potential as a highly selective and novel drug target. Perforin also meets other essential criteria: (i) it is encoded by a single gene, with no functional redundancy;^12^ (ii) the phenotype of perforin-null mice and humans shows that it is essential to CTL/NK killing;^3,12,13^ and (iii) perforin expression is tightly restricted to immune killer cells,^3,4^ so adverse effects of perforin inhibition are predicted to be far fewer than for broad-spectrum drugs such as corticosteroids.

For several years, we have been undertaking a drug discovery program to develop small-molecule inhibitors of perforin as a potential therapy for the preservation of transplanted bone marrow stem cells.^14−17^ Stem cell transplantation is used to treat hematological cancers and nonmalignant disorders such as bone marrow failure and inherited immunodeficiency disorders.^18^ These patients receive pretransplant radio- and chemotherapeutic conditioning regimens; however, residual recipient cell populations can survive and function and contribute to delayed engraftment and graft failure.^19^ Early rejection of mismatched grafts is driven by recipient NK cells, which overwhelmingly use perforin to kill their targets. They can reject greater than 85% of human leukocyte antigen (HLA) mismatched stem cell grafts within 48 h.^20−22^ These cells are both radio-^23^ and immune-suppressant-resistant,^20^ and there are no clinical products that specifically deplete NK cell function. People with a heterozygous perforin deficiency (50–75% perforin activity) demonstrate normal phenotypes, providing genetic evidence to support perforin inhibition as an acceptable method of preventing stem cell rejection.^9,24^ The short-term use of a perforin inhibitor to protect donor stem cells so that they can reach the hematopoietic stem cell niche should allow restoration of bone marrow function with reduced risk of graft failure. Donor stem cell preservation may also accelerate hematopoietic engraftment and reduce susceptibility to infection after stem cell transplant, which is a major cause of morbidity and mortality.^25^

We have previously identified and published a lead series of benzenesulfonamide (BZS) compounds (exemplified by **1** and **2**; Figure 1) that selectively inhibit recombinant perforin and perforin-dependent killing by intact CTL and NK cells, and block perforin activity in mouse and human cells *in vitro* and mice *in vivo.*(14,15) We have also shown that although the BZS compounds are highly effective inhibitors of CTL/NK cell killing, immunity is reestablished when treatment is ceased, demonstrating that perforin inhibition is highly focused and rapidly and completely reversible. In the current publication, we report the detailed pharmacokinetic characterization of eight optimized BZS compounds, identification of the two most suitable exemplars that were subjected to further *in vivo* dose–response experiments, and finally refinement to a single candidate where a pharmacokinetic/pharmacodynamic relationship was developed to inform future *in vivo* efficacy studies.

**Figure 1:**
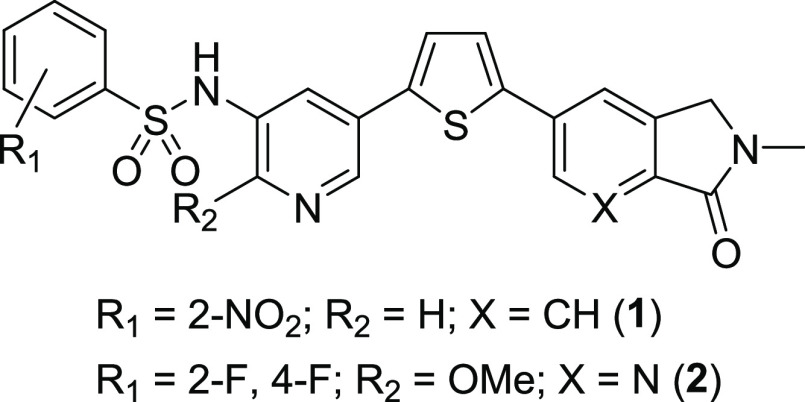
Chemical structures of lead benzenesulfonamide inhibitors.

## Results and Discussion

As an indispensable component of the cytotoxic lymphocyte- and NK-mediated cell death pathway, perforin provides a promising immunosuppression target to address an unmet clinical need in bone marrow stem cell allo-transplantation and potentially other human disorders, including insulin-dependent diabetes, postviral myocarditis, and FVH,^9,10^ as well as therapy-induced conditions such as solid organ rejection and GvHD.^2,11^ We have developed several different structural classes of compounds that inhibit perforin activity *in vitro* and *in vivo*,^14−17,26−31^ and, in this study, focused on eight BZS compounds to establish a pharmacokinetic/pharmacodynamic relationship to inform optimal dosing strategies for future *in vivo* efficacy studies.

### Perforin Inhibition *In Vitro*

We selected eight BZS compounds that potently inhibited the lytic activity of recombinant perforin against Jurkat T leukemia cells and also inhibited the death of K562 leukemic target cells when co-incubated with KHYG-1 NK cells when applied at 10 μM^16,17^ (Table 1). We titrated the eight BZS compounds in a cytotoxicity assay using human KHYG-1 NK cells to determine the concentration required to inhibit 90% of the perforin-dependent cytotoxicity (IC_90_) for each compound. An IC_90_ was used rather than an IC_50_ as we anticipated that elevated suppression of perforin activity is likely required to preserve the transplanted bone marrow stem cells *in vivo*. The IC_90_ values for the eight compounds ranged from 1.86 ± 0.44 to 30.9 ± 10.5 μM (Table 1 and Figure S1). KHYG-1 cells remained viable despite their prolonged (24 h) exposure to the compounds, indicating that the activity was not due to the toxicity of the compounds for the KHYG-1 cells.

**Table 1:**
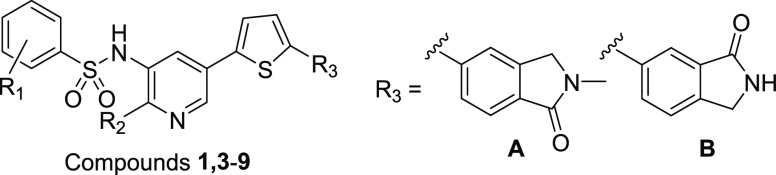
Cytotoxicity of Benzenesulfonamide Perforin Inhibitors

BZS	R_1_	R_2_	R_3_	Jurkat IC_50_ (μM)^a^	KHYG-1 IC_90_ (μM)	KHYG-1 viability (% at 10 μM)
**1**	2-NO_2_	H	A	6.65	5.37 ± 1.10	98.7 ± 1.5
**3**	2,4-DiF	H	A	1.17	16.0 ± 3.6	100
**4**	4-NO_2_	H	A	3.13	19.9 ± 3.7	85.0 ± 12.0
**5**	2,4,6-TriF	H	A	1.76	10.1 ± 1.8	95.1 ± 2.1
**6**	4-CN	H	A	5.17	30.9 ± 10.5	99.3 ± 0.3
**7**	2,4-DiF	H	B	4.15	28.9 ± 6.7	96.0 ± 6.0^b^
**8**	2,4-DiF	F	A	1.99	3.54 ± 0.65	92.0 ± 3.0
**9**	2,4-DiF	Cl	A	1.03	1.86 ± 0.44	94.0 ± 5.3

a Data from refs (16, 17).

b KHYG-1 viability for compound **7** tested at 20 μM.

### Plasma Protein Binding and Pharmacokinetics

Two of the eight compounds had previously been evaluated for plasma protein binding, showing >99.8% binding to proteins in mouse plasma.^17^ To determine if the other six BZS compounds also have high binding to plasma proteins, the level of binding to proteins in mouse plasma was assessed. All six compounds were highly bound to mouse plasma proteins at >99.5%. Mouse plasma pharmacokinetic data has previously been reported for seven of the eight compounds,^16^ and thus was assessed for the remaining compound, **7**. The pharmacokinetic parameter values (*C*_max_ = 235 μM, AUC = 1222 μM·h, *T*_1/2_ = 4.1 h) were within the range of the other seven compounds (Table 2).

**Table 2:** Plasma Protein Binding and Mouse Plasma Pharmacokinetic Parameters for Benzenesulfonamide Perforin Inhibitors

		mouse plasma pharmacokinetics^b^		
BZS	plasma protein binding^a^	*C*_max_ (μM)	AUC (μM·h)	*T*_1/2_ (h)
**1**	99.8	105	415	2.5
**3**	99.89	9.8	220	12
**4**	99.69	64.4	383	2.2
**5**	99.52	124	1019	4.5
**6**	99.77	87.3	642	3.3
**7**	99.97	235	1222	4.1
**8**	99.97	236	2885	6.6
**9**	99.98	149	2364	9.5

a Data for compounds **3** and **7** from ref (17).

b Data for compounds **1**, **3**–**6**, **8**, and **9** from ref (16).

### Perforin Inhibition *In Vivo*

**Figure 2:**
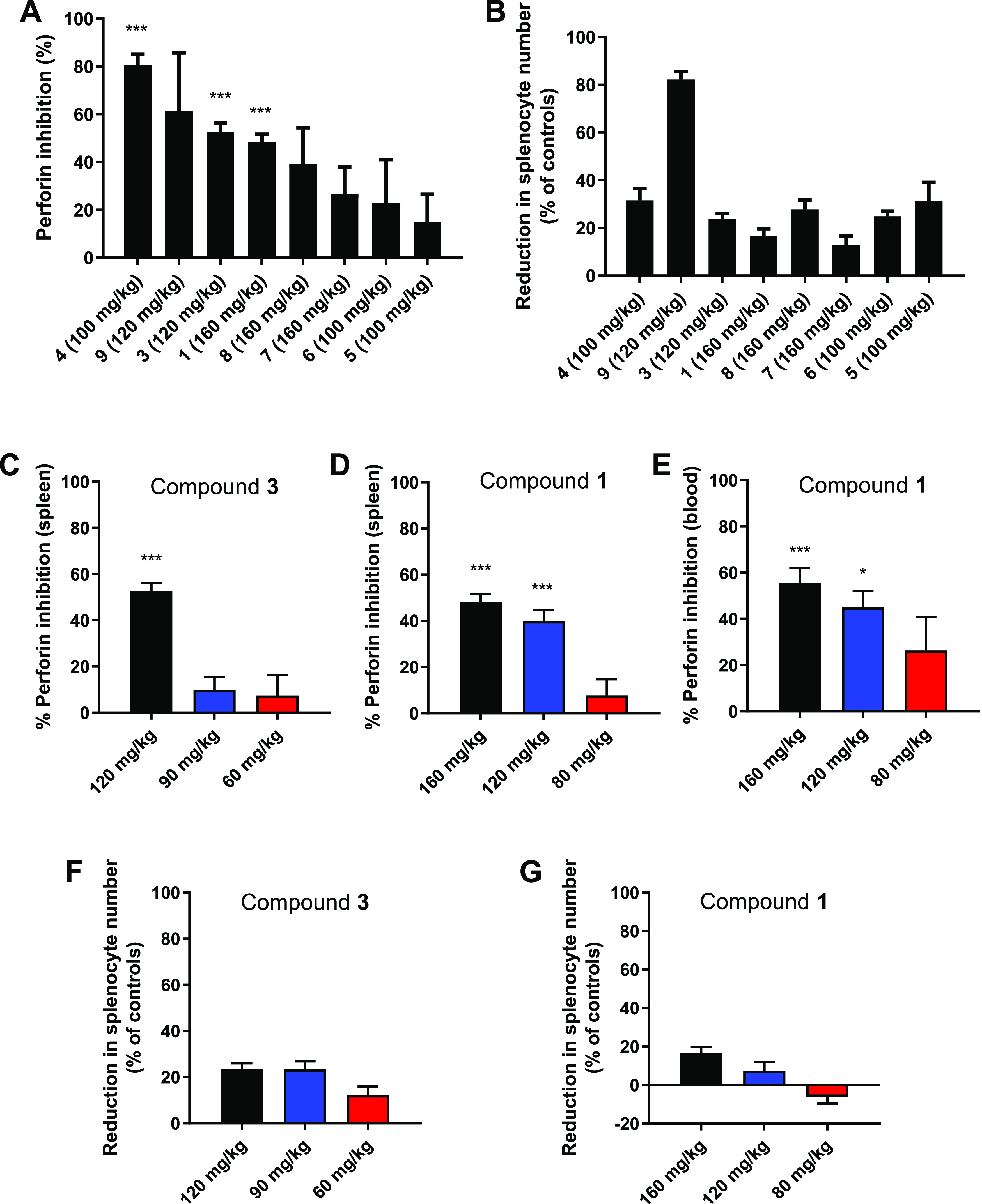
Perforin inhibition and spleen toxicity in mice 24 h after allogeneic bone marrow transplantation. The mice were treated with perforin inhibitors at the indicated doses at 0, 24, and 48 h prior to bone marrow transfer and 18 h afterward. (A) Perforin inhibition in spleen and (B) reduction in mouse spleen size for eight benzenesulfonamide perforin inhibitors. Perforin inhibition in the spleen (C, D) or peripheral blood (E) for multiple dose levels of **3** (C) or **1** (D, E). Reduction in spleen size in mice treated with multiple dose levels of **3** (F) or **1** (G). Bars indicate the mean and standard error of the mean from pooled experiments (5–60 mice). *P < 0.05 and ***P < 0.001 vs untreated controls by one-way ANOVA with Dunnett’s post-test analysis.

All eight compounds proceeded for evaluation of *in vivo* efficacy in a short-term *in vivo* killing assay, in which allogeneic bone marrow cells are the target of CTL/NK-mediated cytotoxicity and syngeneic bone marrow cells serve as an untargeted control. This model was selected as a surrogate assay for graft rejection during allogeneic bone marrow/stem cell transplantation to enable rapid testing of perforin inhibition *in vivo*. Mice were transplanted with a mixture of equal numbers of allogeneic immunologically mismatched and syngeneic immunologically matched bone marrow. The mice were treated with perforin inhibitors at 0, 24, and 48 h prior to and 18 h after cell transfer using a dose at or near the maximum tolerated dose (MTD). Allogeneic cell survival in spleen and peripheral blood was determined 24 h after transplant, using C57BL/6 perforin-deficient mice (100% perforin inhibition) and untreated C57BL/6 mice (0% perforin inhibition) as controls. Three of the compounds, **1**, **3**, and **4**, induced significant increases in allogeneic bone marrow survival in the spleen that were between 48 and 80% of that seen in the perforin-deficient mice, while **9** showed a large but not statistically significant increase (61%) (Figure 2A and Table S1). Perforin inhibition *in vivo* did not follow the same compound rank order as in the *in vitro* KHYG-1 assay, demonstrated by considerable *in vivo* activity of **3** and **4**, despite their moderate IC_90_ values. However, the three compounds with KHYG-1 IC_90_ values ≤5 μM were all effective inhibitors *in vivo*, indicating that the *in vitro* assay is useful for selecting compounds most likely to be active *in vivo*.

Treatment with perforin inhibitors *in vivo* was also associated with a reduction in splenocyte cellularity, which was typically 10–30% of the control cell count but was much higher for **9** (Figure 2B). The two compounds (**1** and **3**) that most effectively inhibited perforin function with low reductions in splenic cellularity were next evaluated for a dose–response relationship at 100, 75, and 50% of MTD. Compound **3** showed significant perforin inhibition in the spleen, with 53% of the effect seen in perforin-deficient mice at 120 mg/kg, but a nonsignificant inhibition of 10 and 7% at 90 and 60 mg/kg, respectively (Figure 2C). Compound **1** induced significant perforin inhibition in the spleen of 48 and 40% at 160 and 120 mg/kg, respectively, but a nonsignificant 7% inhibition at 80 mg/kg (Figure 2D). Allogeneic bone marrow survival in blood was also determined for the three dose levels of **1** with a significant increase in perforin inhibition relative to controls of 55 and 45% at 160 and 120 mg/kg, respectively, and a nonsignificant 26% inhibition at 80 mg/kg (Figure 2E). All dose levels of **1** and **3** were well tolerated and produced only minor reductions in splenocyte number (Figure 2F,G) that were not definitively dose-related.

Splenic atrophy may occur as a direct treatment-related effect or indirectly subsequent to bodyweight loss.^32^ Spleen size does normally vary so is not a reliable indicator of splenic dysfunction;^33^ however, severe atrophy can prevent the normal function of the spleen and indicate systemic immunotoxicity.^34^ Given that reduced splenic cellularity had not been noted in other *in vivo* mouse disease models using **1**,^14,15^ we further investigated the impact of **1** on mouse spleen using extended dosing strategies on unmanipulated naïve mice and assessing changes in bodyweight, splenocyte, and blood cell counts and leukocyte frequencies in the spleen. The mice were treated with either 100 or 150 mg/kg of **1** given by intraperitoneal injection twice daily for up to 7 days. Neither dosing schedule resulted in weight loss, change in spleen or blood cell counts or any reduction in splenic CD4^+^ or CD8^+^ T cells and CD19^+^ B cells or NK1.1^+^ NK cells, compared to vehicle control (Table 3). This suggested that the reduced splenocyte counts we observed in the *in vivo* perforin inhibition assay were likely related to bone marrow transplantation. This was consistent with the fact that we had not observed similar effects with compound **1** in other *in vivo* settings.^15^ Although both compounds **1** and **3** showed significant increases in allogeneic bone marrow cell survival and only minor reductions in splenocyte number, we ultimately selected compound **1** for the evaluation of a PK/PD relationship because it produced a more linear dose-dependent increase in allogeneic bone marrow survival than **3**.

**Table 3:** Mouse Bodyweight, Spleen, and Blood Counts for Compound **1**

	vehicle (bid ×7; *n* = 3)	100 mg/kg **1** (bid ×7; *n* = 6)	150 mg/kg **1** (bid ×3; *n* = 3)
% change in bodyweight	–7 ± 7	–5 ± 2	–5.1 ± 5.9
spleen: total cell count (×10^8^)	0.34 ± 0.07	0.39 ± 0.08	0.41 ± 0.46
spleen: % CD3^+^CD4^+^	8.8 ± 6	7.9 ± 3.3	5.6 ± 3.8
spleen: % CD3^+^CD8^+^	7.8 ± 2.9	9.1 ± 2.6	10.6 ± 3.4
spleen: % NK1.1^+^	2.5 ± 0.7	2.3 ± 0.6	5.0 ± 0.7
spleen: % CD19^+^	54.3 ± 5.9	45.4 ± 10.1	41.4 ± 5.3
white blood cell (×10^9^/L)	4.0^a^	6.1 ± 2.0^b^	6.0 ± 0.4
red blood cell (×10^12^/L)	9.7^a^	9.9 ± 0.3^b^	9.5 ± 0.9
platelets (×10^9^/L)	951^a^	1041.5 ± 0.3^b^	1163 ± 149

a *n* = 1.

b *n* = 2.

### Compound **1** Mouse Pharmacokinetics

**Figure 3:**
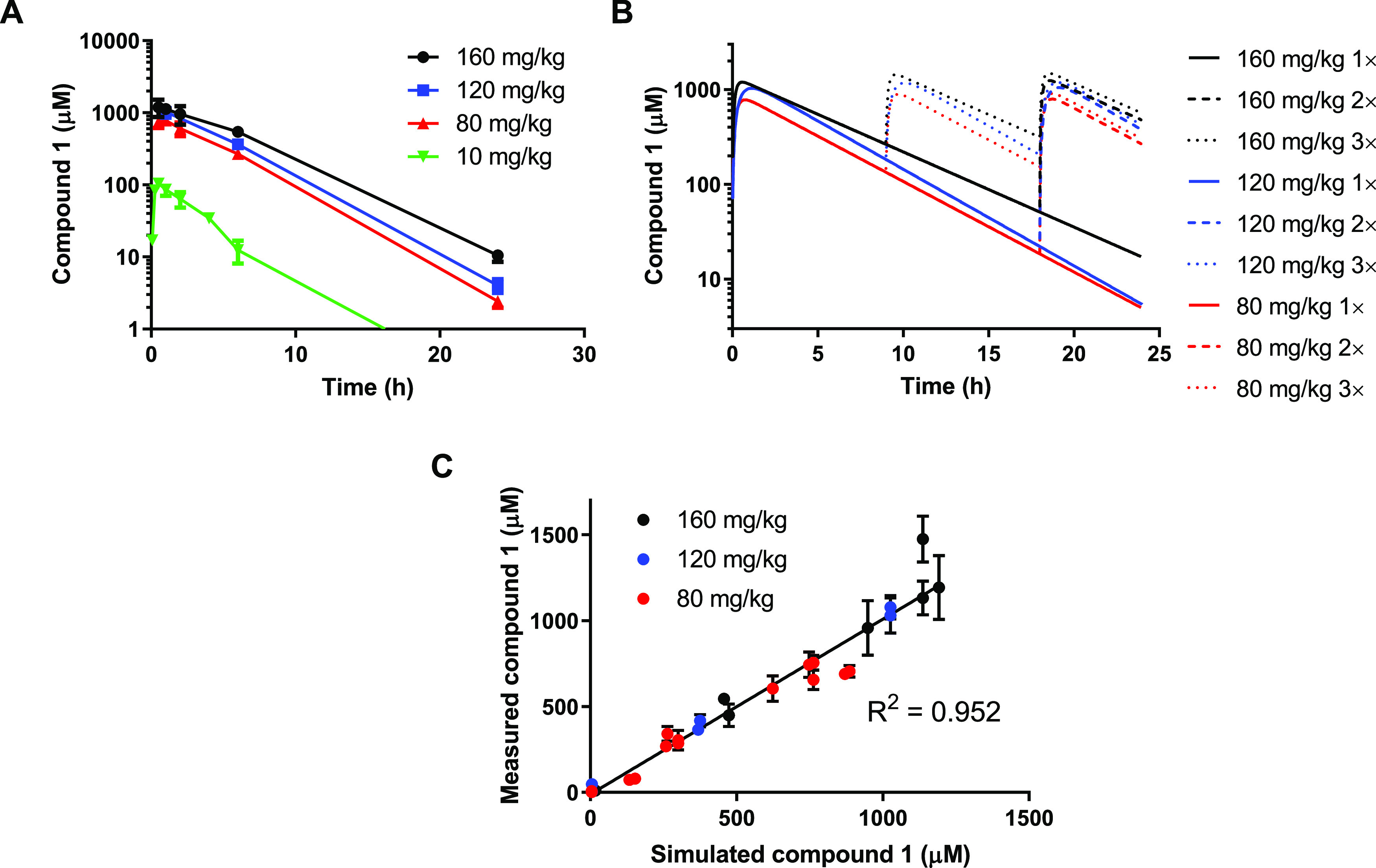
Compound **1** mouse pharmacokinetics. (A) Plasma concentration–time profiles of multiple dose levels of **1** by single dose. (B) Simulated plasma concentration–time profiles of different multiple dose schedules of **1** based on a one-compartment pharmacokinetic model. (C) Comparison of measured concentrations of **1** after one dose at 0 h (1×); two doses at 0 and 18 h (2×); or three doses at 0, 9, and 18 h (3×) with simulated data generated at the same time point using a one-compartment pharmacokinetic model. Experimental datapoints represent the mean and standard error of three animals.

For determination of plasma concentrations of **1**, C57BL/6 mice were treated with a single dose of **1** at four different dose levels (160, 120, 80, and 10 mg/kg) and blood samples were collected at multiple timepoints after dosing. Overall, the pharmacokinetics followed a dose-dependent relationship that was close to linear (Figure 3A). High concentrations were achieved initially with *C*_max_ values at the upper dose levels in the mM range, but these declined to low or negligible concentrations by 24 h with an elimination half-life of between 2.4 and 3.4 h observed across the different dose levels (Table 4). The concentration–time data were best fitted to a one-compartment pharmacokinetic model that was used to simulate different dosing schedules to predict optimal dose levels and schedules of compound **1** administration to maintain the maximum concentrations throughout the 24 h transplant period in our *in vivo* bone marrow survival assay (Figure 3B). To confirm the accuracy of the simulations, C57BL/6 mice were treated intraperitoneally with 80, 120, or 160 mg/kg dose of **1** and the resulting plasma concentrations were evaluated at multiple time points within a 24 h period after one (0 h), two (0, 18 h) or three (0, 9 h, 18 h) doses. The measured concentrations correlated closely with the predicted concentrations from the simulations, regardless of the number of doses administered (Figure 3C), indicating that **1** plasma pharmacokinetics can be accurately fitted using a one-compartment model.

**Table 4:** Mouse Plasma Pharmacokinetic Parameters for Compound **1**

dose (mg/kg)	*C*_max_ (μM)	AUC_0–24_ (μM·h)	*T*_1/2_ (h)
160	1193	8936	3.36
120	1030	7317	2.85
80	782	5442	2.85
10	105	413	2.38

### Compound **1***In Vivo* Efficacy

**Figure 4:**
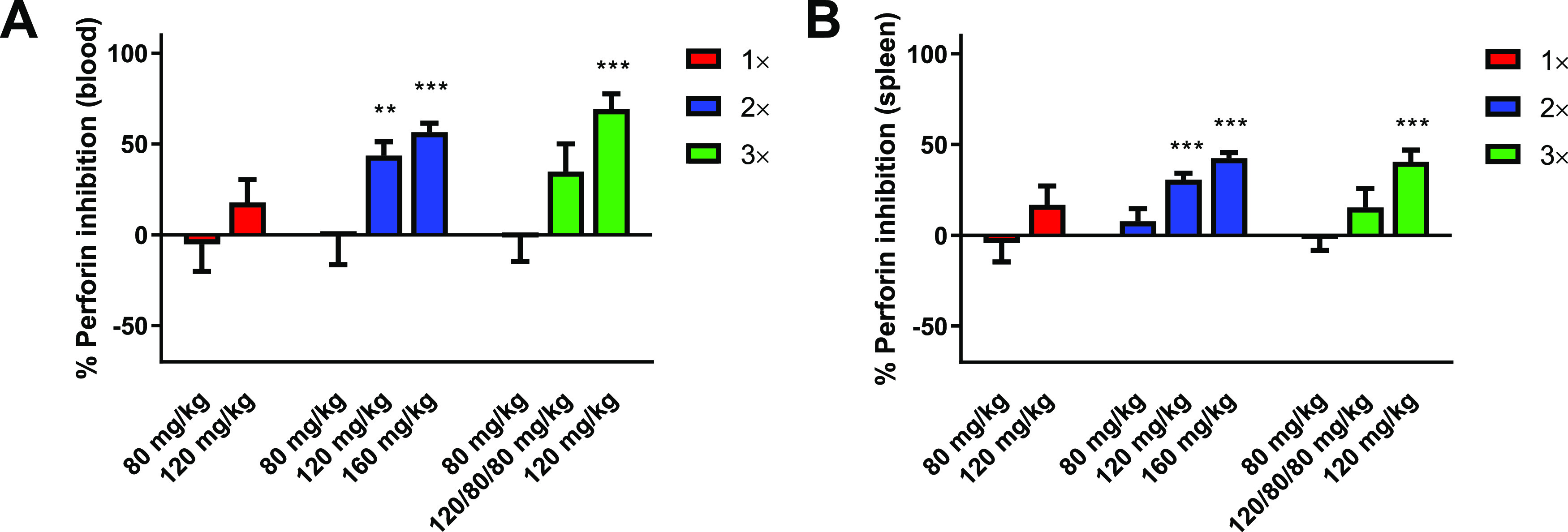
Perforin inhibition in peripheral blood (A) and spleen (B) in mice 24 h after allogeneic bone marrow transplantation. The mice were treated with compound **1** at one dose (1×), two dose (2×), and three dose (3×) schedules at 0 h; 0 and 18 h; and 0, 9, and 18 h, respectively, starting immediately prior to transfer. Bars indicate the mean and standard error of the mean for pooled experiments (9–58 mice). **P < 0.01 and ***P < 0.001 vs untreated controls by one-way ANOVA with Dunnett’s post-test analysis.

Next, we tested multiple schedules and doses of compound **1** in the *in vivo* bone marrow transfer assay to determine if more frequent dosing could enhance efficacy. The short half-life of **1** provided an opportunity to alter the dose and schedule to achieve varied concentrations within the 24 h transplant window. C57BL/6 mice transplanted as described above were treated with one dose of **1** at 0 h (immediately prior to transplant); two doses at 0 and 18 h; or three doses at 0, 9, and 18 h at 80, 120, or 160 mg/kg. Unlike the previous study, doses were not administered at 24 and 48 h pretransplant, since concentrations at 24 h after dosing were low and accumulation minimal. However, to increase sample size and reduce animal use, data from the previous study with pretransplant dosing were pooled with the two dose data as these groups had identical simulated PK parameters. As per the data in Figure 2D,2E, doses of 120 and 160 mg/kg were able to promote survival of allogeneic bone marrow transplant in the peripheral blood (Figure 4A) and spleen (Figure 4B), with two and three dose schedules significantly improving bone marrow cell survival; a three dose schedule at 160 mg/kg was not evaluated due to expected low tolerance (Table S2).

### PK/PD Relationship for Compound **1** Efficacy

**Figure 5:**
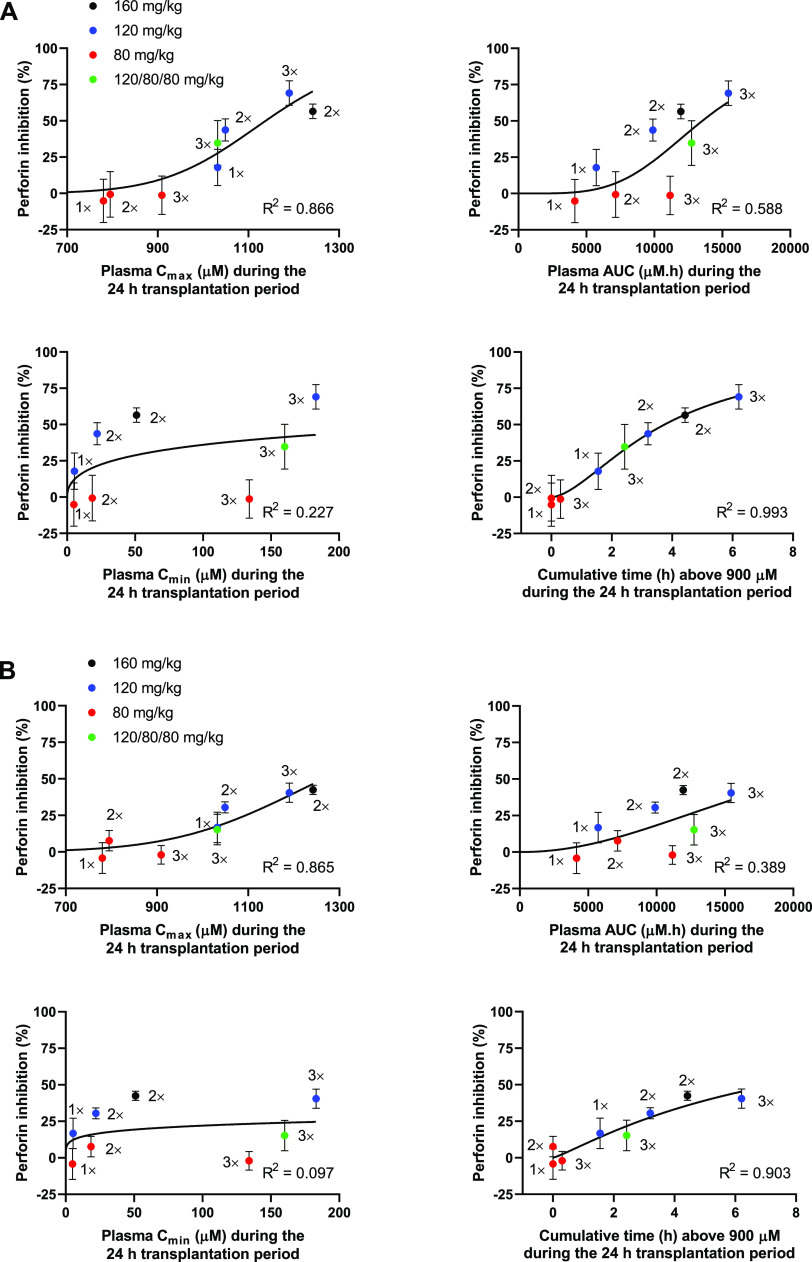
Pharmacokinetic/pharmacodynamic relationship for compound **1** efficacy. Comparison of % perforin inhibition in peripheral blood (A) and spleen (B) in the *in vivo* bone marrow transfer assay with compound **1** plasma *C*_max_, plasma AUC, plasma *C*_min_, and cumulative time that concentrations of **1** exceeded 900 μM for multiple dose levels and schedules (1×, one dose at 0 h; 2×, two doses at 0 and 18 h; 3×, three doses at 0, 9, and 18 h) of **1**. Datapoints represent the mean and standard error of the mean for 9–58 mice; curved lines and regression analysis indicate fit by the sigmoid *E*_max_ model. Pharmacokinetic values were determined from the one-compartment model simulations.

To determine the plasma drug concentrations required to achieve *in vivo* efficacy and establish the PK parameters that can best predict efficacy, a PK/PD relationship was determined for compound **1** using the PK and *in vivo* efficacy data generated for the different doses and schedules above. The relationship between efficacy (% perforin inhibition in peripheral blood and spleen) and pharmacokinetics was determined for different pharmacokinetic properties (Figure 5A,B). Inhibition of perforin was found to increase with higher *C*_max_ values (blood: *R*^2^ = 0.866, spleen: *R*^2^ = 0.865), but there was no strong correlation with total AUC (blood: *R*^2^ = 0.588, spleen: *R*^2^ = 0.389) or C_min_ (blood: *R*^2^ = 0.227, spleen: *R*^2^ = 0.097) during the 24 h transplantation window. The strongest correlation in both peripheral blood and spleen was observed between perforin inhibition and the time concentrations remained above a high level, arbitrarily chosen as 900 μM (blood: *R*^2^ = 0.993, spleen: *R*^2^ = 0.903), which was the only correlation able to explain why three doses of 120 mg/kg were highly active, while three doses of 80 mg/kg were ineffective. This PK/PD relationship suggests that high concentrations of **1** (>900 μM) need to be continuously maintained for optimal perforin inhibition in the *in vivo* mouse model. Simply maintaining the concentrations above a minimal level without reaching very high levels was not sufficient for perforin inhibition, as exemplified by three doses at 80 mg/kg. Achieving a high initial *C*_max_ with a dose of 120 mg/kg followed by two doses of 80 mg/kg only resulted in moderate perforin inhibition, further suggesting that high concentrations were not achieved for long enough to obtain effective perforin inhibition.

### Compound **1** Protein Binding

Since compound **1** has high binding to plasma proteins (Table 2), there is potential for saturation of binding to plasma proteins at very high concentrations, which could help explain why time above 900 μM was strongly associated with efficacy. To investigate if saturation of plasma protein binding can occur with high concentrations of compound **1**, the plasma protein binding of 10, 100, 500, and 1000 μM of **1** was determined by equilibrium dialysis. Compound **1** was highly plasma protein-bound at all concentrations, but there was a small reduction in plasma protein binding at 1000 μM (Table 5), resulting in a 4-fold increase in free drug concentrations between 500 and 1000 μM (free drug concentration = 0.95 and 3.8 μM, respectively). For comparison of unbound concentrations in plasma with unbound potency *in vitro*, binding of **1** to proteins in the media used in the KHYG-1 cell *in vitro* assay was also determined. The free fraction in the KHYG-1 cell media was determined to be 0.144, allowing us to estimate an unbound *in vitro* IC_90_ of **1** in KHYG-1 cells of 0.77 μM, based on an IC_90_ value of 5.37 ± 1.10 μM in KHYG-1 cell media (Table 1).

**Table 5:** Plasma Protein Binding for Compound **1**

concentration (μM)	plasma protein binding (%)	unbound concentration (μM)
10	99.8	0.02
100	99.94	0.06
500	99.81	0.95
1000	99.62	3.8

Without saturation of plasma protein binding, at a free fraction of 0.002 (i.e., 99.8% protein-bound), total plasma concentrations of ∼350 μM are required to achieve unbound plasma concentrations equivalent to the *in vitro* unbound IC_90_ of compound **1**. However, maintaining concentrations at 350 μM is not sufficient for *in vivo* efficacy, as the 80 mg/kg three-dose schedule achieved 350 μM for a slightly longer duration (14.9 h) than two doses of 120 (12.0 h) or 160 (13.4 h) mg/kg, but was less active. Therefore, based on our PK/PD relationship, where the strongest relationship was based on time above 900 μM, to maximize the efficacy of our perforin inhibitors, we estimate that we need to maintain concentrations above 3× the unbound IC_90_ for as long as possible within the transplantation window. Given the high concentrations required, there is potential for saturation of plasma protein binding to occur, where small dose increments could cause large increases in unbound concentrations.^35^ Indeed, some evidence of saturated plasma protein binding was observed with 1000 μM of compound **1**; however, we cannot be certain this is not due to experimental variation as a result of poor solubility when spiking plasma at pH 7.4 with such high concentrations of the compound, and so have not incorporated plasma protein binding saturation into our PK/PD relationship. Additionally, the saturation of plasma protein binding does not help to explain the difference in effective concentration between *in vitro* and *in vivo* studies, as the effective *in vivo* unbound concentration increases beyond 3× the *in vitro* unbound IC_90_ with the saturation of plasma protein binding above 900 μM.

The outcome of our PK/PD relationship informs optimal dosing strategies for future *in vivo* efficacy studies. The short elimination half-life of BZS compounds means they require frequent administration at high doses to maintain sufficiently high concentrations to induce perforin inhibition for as long as possible. This approach may be suitable for a short-term assay used here with a 24 h transplantation window; however, for longer-term assays, alternative strategies may be required. Patients receiving allogeneic bone marrow transplantation are hospitalized and require an intravenous line for administration of all medications.^36^ As a result, reliable intravenous access in the form of a central venous port is always available. In this context, IV therapy is a suitable approach for this indication as it will enable drug administration in the critical 4–5 days after transplantation to block rejection and allow increased stem cell numbers to survive and reach the bone marrow stem cell niche. Thus, drug administered by periodic short-term infusions could provide and maintain the high concentrations needed for perforin inhibition. Additionally, we are now focusing on developing BZS analogues with longer elimination half-lives in excess of 8 h for oral dosing to ensure we can maintain concentrations above 3× the unbound IC_90_ for the full 24 h transplantation window with only a single daily dose.

These studies have demonstrated proof of concept for the use of BZS perforin inhibitors in the preservation of hematopoietic stem cells transplanted across a complete MHC mismatch. Incorporation of this PK/PD relationship into our drug discovery program will assist our ongoing efforts to identify a novel perforin inhibitor with optimal *in vivo* efficacy to progress toward clinical evaluation.

## Materials and Methods

### Perforin Inhibitors

The following compounds were synthesized in house at the Auckland Cancer Society Research Centre: The University of Auckland (Auckland, New Zealand): (*N*-{5-[5-(2-methyl-1-oxo-2,3-dihydro-1*H*-isoindol-5-yl)-2-thienyl]-3-pyridinyl}-2-nitrobenzenesulfonamide) (**1**), (2,4-difluoro-*N*-{5-[5-(2-methyl-1-oxo-2,3-dihydro-1*H*-isoindol-5-yl)-2-thienyl]-3-pyridinyl}benzenesulfonamide) (**3**), (*N*-{5-[5-(2-methyl-1-oxo-2,3-dihydro-1*H*-isoindol-5-yl)-2-thienyl]-3-pyridinyl}-4-nitrobenzenesulfonamide) (**4**), (2,4,6-trifluoro-*N*-{5-[5-(2-methyl-1-oxo-2,3-dihydro-1*H*-isoindol-5-yl)-2-thienyl]-3-pyridinyl}benzenesulfonamide) (**5**), (4-cyano-*N*-{5-[5-(2-methyl-1-oxo-2,3-dihydro-1*H*-isoindol-5-yl)-2-thienyl]-3-pyridinyl}benzenesulfonamide) (**6**), (2,4-difluoro-*N*-{5-[5-(3-oxo-2,3-dihydro-1*H*-isoindol-5-yl)-2-thienyl]-3-pyridinyl}benzenesulfonamide) (**7**), (2,4-difluoro-*N*-{2-fluoro-5-[5-(2-methyl-1-oxo-2,3-dihydro-1*H*-isoindol-5-yl)-2-thienyl]-3-pyridinyl}benzenesulfonamide) (**8**), (*N*-{2-chloro-5-[5-(2-methyl-1-oxo-2,3-dihydro-1*H*-isoindol-5-yl)-2-thienyl]-3-pyridinyl}-2,4-difluorobenzenesulfonamide) (**9**) as previously described.^16,17^ Chemical purity exceeded 98% by HPLC.

### KHYG-1 Cytotoxicity Assay

The inhibition of the cytotoxic activity of KHYG-1 NK cells on K562 target cells was determined as described previously.^14^ Briefly, compounds were added to KHYG-1 cells at 6–7 concentrations ranging from 20 μM to 312.5 nM in 2-fold dilutions in RPMI medium (Thermo Fisher Scientific, Waltham, MA) supplemented with 0.1% bovine serum albumin (BSA; Sigma-Aldrich, St. Louis, MO) for 20 min. ^51^Cr-labeled K562 leukemia target cells were added to KHYG-1 cells at an effector/target ratio of 2:1 and incubated at 37 °C for 4 h. ^51^Cr release was assayed using a Harvesting Press (Skatron Instruments, Lier, Norway), and radioactivity was estimated on a Wallac Wizard 1470 Automatic γ counter (Turku, Finland). The IC_90_ values were calculated by fitting a four-parameter logistic sigmoidal dose–response curve to the data using Prism version 8 (GraphPad, San Diego, CA). Each IC_90_ value represents the mean of at least three independent experiments, where six to seven concentrations were tested in triplicate for each compound.

### KHYG-1 Viability

The toxicity of perforin inhibitors toward KHYG-1 NK cells was determined as described previously.^14^ KHYG-1 cells were preincubated with compounds in the same manner as the KHYG-1 cytotoxicity assay prior to the addition of RPMI with 0.1% BSA and incubation at 37 °C for 4 h. The cells were then washed and resuspended in complete medium and incubated for a further 24 h at 37 °C, after which cell viability was assessed by Trypan Blue (Thermo Fisher Scientific) exclusion.

### Protein Binding

The plasma protein binding of the compounds was determined by equilibrium dialysis (12–14 kDa cutoff membrane). Pooled CD1 mouse plasma (Innovative Research, Novi, MI) (pH adjusted to 7.4, 100 μL) was spiked with test compounds at a final concentration of 10 μM and added to one side of an HTDialysis (Gales Ferry, CT) apparatus with an equal volume of PBS (100 mM, pH 7.4) added to the other side. The dialysis unit was covered with an adhesive sealing film to prevent evaporation of the contents and placed in an incubator (Innova-42, New Brunswick Scientific) maintained at 37 °C with gentle mixing (80 rpm) for 6 h. Next, a 10 μL sample was aspirated from the plasma side and mixed with 90 μL of fresh PBS, whereas a 90 μL sample from the buffer side was removed and combined with 10 μL of drug-free plasma to ensure uniform matrix composition. Ice-cold acetonitrile (300 μL; Merck Millipore, Burlington, MA) containing internal standard (a BZS analogue of similar chemical structure) was added to each sample to precipitate protein and was vortexed for 2 min and centrifuged for 5 min at 13 000 rpm. The supernatant was diluted with 0.01% formic acid in ultrapure water (Milli-Q) (1:1) and quantitated by liquid chromatography–tandem mass spectrometry (LC-MS/MS). Each compound was tested in triplicate alongside control compounds known to have low (e.g., lidocaine) and high (e.g., amitriptyline) plasma protein binding. For media binding studies, the same assay was used, but RPMI with 0.1% BSA was used in place of CD1 mouse plasma.

### Mouse Pharmacokinetic Studies

Male C57BL/6 mice (18–25 g) were supplied and housed under controlled temperature and humidity at the Vernon Jansen Unit of the University of Auckland. The mice had access to a pelleted diet and water *ad libitum*. All animal experimentation followed protocols approved by the University of Auckland Animal Ethics Committee (approval #001781). Dosing solutions were prepared in 20% hydroxypropyl-β-cyclodextrin (Sigma-Aldrich). The mice received one to three doses by intraperitoneal route and were euthanised at predetermined time points (*n* = 3 mice per time point at 0.083, 0.25, 0.5, 1, 2, 4, 6, and 24 h for 10 mg/kg **1** and **7**; 0.5, 1, 2, 6, and 24 h for 80 and 160 mg/kg **1**; and 1, 6, and 24 h for 120 mg/kg **1**). Blood samples (∼300 μL) were obtained through cardiac bleed and collected in ice-cold K_2_-EDTA tubes (BD, Franklin Lakes, NJ). Plasma was separated by centrifugation at 6500 rpm for 5 min and stored at −80 °C. On the day of analysis, plasma samples were thawed on ice and the analytes were extracted by mixing 10 μL of aliquots with four volumes of ice-cold acetonitrile/methanol (1:1 v/v) containing internal standard. The samples were kept on ice for 10 min to ensure complete protein precipitation, vortexed for 2 min, and centrifuged at 13 000 rpm for 5 min at room temperature. The clear supernatant was then diluted (1:1 v/v) with 40 μL of 0.01% formic acid in Milli-Q water prior to analysis by LC-MS/MS.

### LC-MS/MS Analysis

Compound concentration in biological matrices was measured by LC-MS/MS using a 6410b triple-quadrupole mass spectrometer (Agilent, Santa Clara) equipped with a multimode ionization source. The optimized mass detector conditions, namely, ionization polarity, precursor product ions, fragmentor voltage, and collision energy, were manually determined by infusing a 5 μM solution of the pure compound into the ion source. Chromatographic separation was achieved using a Zorbax SB-C18, 50 × 2.1 mm^2^, 5 μm column (Agilent), and an Agilent 1100 series HPLC system. The mobile phase used was (A) 0.01% formic acid in Milli-Q water and (B) 80% acetonitrile containing 0.01% formic acid in ultrapure water at a flow rate of 0.6 mL/min. A gradient elution was used over 5 min, and the flow rate was maintained at 0.6 mL/min throughout the gradient. The column oven temperature and the autosampler temperature were set to 35 and 4 °C, respectively. The quantitation was achieved with MS/MS detection in electrospray ionization in negative-ion mode. The instrument multimode source parameters were set as follows: gas temperature, 325 °C; vaporizer, 250 °C; gas flow, 5 L/min; and nebulizer, 45 psi. The ion spray voltage was set at 3000 V. Detection of the compounds was carried out in multiple reaction monitoring mode by monitoring the *m*/*z* transitions of precursor to selective product ion. For each compound, fragmentor voltage and collision energy were optimized to achieve maximum abundance. Quadrupoles Q1 and Q3 were set to unit resolution.

Test samples were diluted up to 100-fold in blank plasma as necessary and quantitated against a calibration curve of known analyte concentrations ranging from 3 nM to 30 μM in an appropriate matrix and quality controls at 0.03, 0.3, 3, and 30 μM. Pharmacokinetic parameters were evaluated using noncompartmental analysis and pharmacokinetic simulations using one-compartment analysis on Phoenix WinNonlin version 6.2 (Certara, Princeton, NJ). Multiple-dose simulations were generated from parameters derived from single-dose simulations.

### *In Vivo* Perforin Inhibition

Compounds were tested for *in vivo* efficacy as described previously.^14^ Age- (8–12 weeks) and sex-matched wild-type (H-2D^b^, CD45.2^+^) or perforin-deficient C57BL/6 mice (C57BL/6-*Prf1*^*tm1Sdz*^/J; H-2D^b^, CD45.2^+^) received 12 ×10^6^ bone marrow cells from both allogeneic BALB/c (H-2D^d^) MHC-*mis*matched and syngeneic C57BL/6 (H-2D^b^) MHC-matched mice (24 × 10^6^ cells total). The wild-type recipient mice were treated with perforin inhibitor compounds by intraperitoneal injection in 20% hydroxypropyl-β-cyclodextrin vehicle (10 mL/kg), or with vehicle alone; perforin-deficient mice remained untreated. The mice were euthanized 24 h after bone marrow transfer for peripheral blood mononuclear cell (PBMC) and spleen collection.

Throughout the study, we utilized two strategies to distinguish between recipient, donor allogeneic, and donor syngeneic cells based on either fluorescent labeling prior to transplant or congenic marker expression (CD45.1/CD45.2). In the fluorescent dye approach, allogeneic wild-type BALB/c (H-2D^d^, CD45.2^+^) bone marrow was labeled with 1 μM carboxyfluorescein succinimidyl ester (CFSE) prior to mixing with syngeneic bone marrow from C57BL/6 congenic mice (CD45.1^+^). Twenty-four hours after cell transfer, the harvested cells were stained with anti-CD45.1-phycoerythrin and anti-TER119-allophycocyanin monoclonal antibodies (BioLegend, San Diego, CA). TER119 was used for erythroid exclusion prior to the identification of allogeneic (CFSE^+^) and syngeneic (CD45.1^+^) donor cells. Using a second purely congenic marker approach, allogeneic BALB/c congenic (H-2D^d^, CD45.1^+^) bone marrow was mixed with syngeneic C57BL/6 double congenic (H-2D^b^, CD45.1^+^CD45.2^+^) bone marrow prior to transfer. The harvested cells were stained with anti-CD45.1-phycoerythrin, anti-CD45.2-phycoerythrin-cyanine-7, and anti-TER119-allophycocyanin monoclonal antibodies (BioLegend, San Diego, CA). TER119 was used for erythroid exclusion, prior to identification of allogeneic donor (CD45.1^+^), syngeneic donor (CD45.1^+^ CD45.2^+^), and recipient (CD45.2^+^) cells. Dead cells were excluded using 7-aminoactinomycin D. Samples were acquired with an LSR Fortessa (BD Biosciences) and analyzed using FlowJo version 9 software (BD Biosciences). Prism version 9 (GraphPad) was used to calculate CTL indices as the percentage survival of syngeneic donor cells divided by the percentage survival of allogeneic donor cells. Percentage perforin inhibition was derived by normalization of CTL indices within each experiment to perforin-deficient mice (100% inhibition) and vehicle-treated mice (0% inhibition) prior to pooling across experiments. *In vivo* perforin inhibition animal experiments were approved by the Animal Ethics Committee of the QIMR Berghofer Medical Research Institute.

### Mouse Spleen and Blood Cellularity

For the determination of splenic cell counts, mouse spleens were harvested and the cells dispersed in media by gentle teasing and then centrifuged at 500*g*. Cell pellets were resuspended in media and total spleen cell counts were performed manually using a standard hemocytometer and light microscopy. Lymphocyte subsets were quantified by flow cytometry using the following antibody reagents: BV605 rat anti-mouse CD3 clone 17A2 (#564009, BD Biosciences); BV786 rat anti-mouse CD4 clone RM4–5 (#563727, BD Biosciences); BV711 rat anti-mouse CD8a clone 53-6.7 (#100748, BioLegend); PE-Cy7 mouse anti-mouse NK1.1 clone PK136 mouse (#552878, BD Biosciences); APC rat anti-mouse CD19 clone 1D3 (#550992, BD Biosciences). Peripheral blood cell counts and spleen white blood cell counts were determined on a Beckman Coulter Ac.T diff hematology analyzer.

### Statistical Analysis

The normality of *in vivo* perforin inhibition data was checked by Shapiro–Wilk and D’Agostino–Pearson normality tests. Statistical tests were performed by one-way ANOVA with multiple comparisons made to the vehicle-treated group and corrected using Dunnett’s post-test analysis. A comparison of measured and simulated plasma concentrations was analyzed by linear regression. Efficacy data were correlated to pharmacokinetic data using a sigmoid *E*_max_ model and regression analysis. All statistical analyses were conducted using Prism v8 or 9.

## Supporting Information Available

Representative KHYG-1 cytotoxicity plots for compounds **1**–**9** (Figure S1); *in vivo* perforin inhibition assay data and sample size for the eight BZS compounds (Table S1); and *in vivo* perforin inhibition assay data and sample size for the PK/PD relationship for compound **1** (Table S2) (PDF)

## Abbreviations

AUC: area under the concentration–time curve
BSA: bovine serum albumin
BZS: benzenesulfonamide
CTL: cytotoxic T lymphocyte
FVH: fulminant viral hepatitis
GvHD: graft versus host disease
HLA: human leukocyte antigen
LC-MS/MS: liquid chromatography–tandem mass spectrometry
MHC: major histocompatibility complex
MTD: maximum tolerated dose
NK: natural killer
PBMC: peripheral blood mononuclear cell
PK/PD: pharmacokinetic/pharmacodynamic.
*T*_1/2_: elimination half-life
