# Supplementary material for: Preclinical Activity and Pharmacokinetic/Pharmacodynamic Relationship for a Series of Novel Benzenesulfonamide Perforin Inhibitors
Source: ACS Pharmacol Transl Sci. 2022 May 31;5(6):429–39. doi: 10.1021/acsptsci.2c00009 (PMC9194936; doi:10.1021/acsptsci.2c00009)
Supplement: Supplementary file 1 — pt2c00009_si_001.pdf [file pt2c00009_si_001.pdf]

Supporting Information:

Preclinical activity and pharmacokinetic/ pharmacodynamic relationship for a series of novel benzenesulphonamide perforin inhibitors

Kate H. Gartlan[1‡], Jagdish K. Jaiswal[2,3‡], Matthew R. Bull[2,3], Hedieh Akhlaghi[4], Vivien R. Sutton[4,5], Kylie A. Alexander[1], Karshing Chang[1], Geoffrey R. Hill[1,6], Christian K. Miller[2,3], Patrick D. O'Connor[2,3], Jiney Jose[2,3], Joseph A. Trapani[4,5], Susan A. Charman[7], Julie A. Spicer[2,3,8], Stephen M. F. Jamieson[2,3,8].*

[1]QIMR Berghofer Medical Research Institute, 300 Herston Road, Herston, Queensland 4006, Australia.

[2]Auckland Cancer Society Research Centre, Faculty of Medical and Health Sciences, The University of Auckland, Private Bag 92019, Auckland 1142, New Zealand.

[3]Maurice Wilkins Centre for Molecular Biodiscovery, The University of Auckland, Private Bag 92019, Auckland 1142, New Zealand.

[4]Cancer Immunology Program, Peter MacCallum Cancer Centre, 305 Grattan Street, Melbourne, Victoria 3000, Australia.

[5]Sir Peter MacCallum Department of Oncology, The University of Melbourne, Parkville, Victoria 3052, Australia.

[6]Clinical Research Division, Fred Hutchinson Cancer Research Center, Seattle, WA 98109, USA

[7]Centre for Drug Candidate Optimisation, Monash Institute of Pharmaceutical Sciences, Monash University, 381 Royal Parade, Parkville, VIC, 3052, Australia.

[8]Department of Pharmacology and Clinical Pharmacology, Faculty of Medical and Health Sciences, The University of Auckland, Private Bag 92019, Auckland 1142, New Zealand.

*Corresponding author: Stephen Jamieson: s.jamieson@auckland.ac.nz; +64 9 923 9141

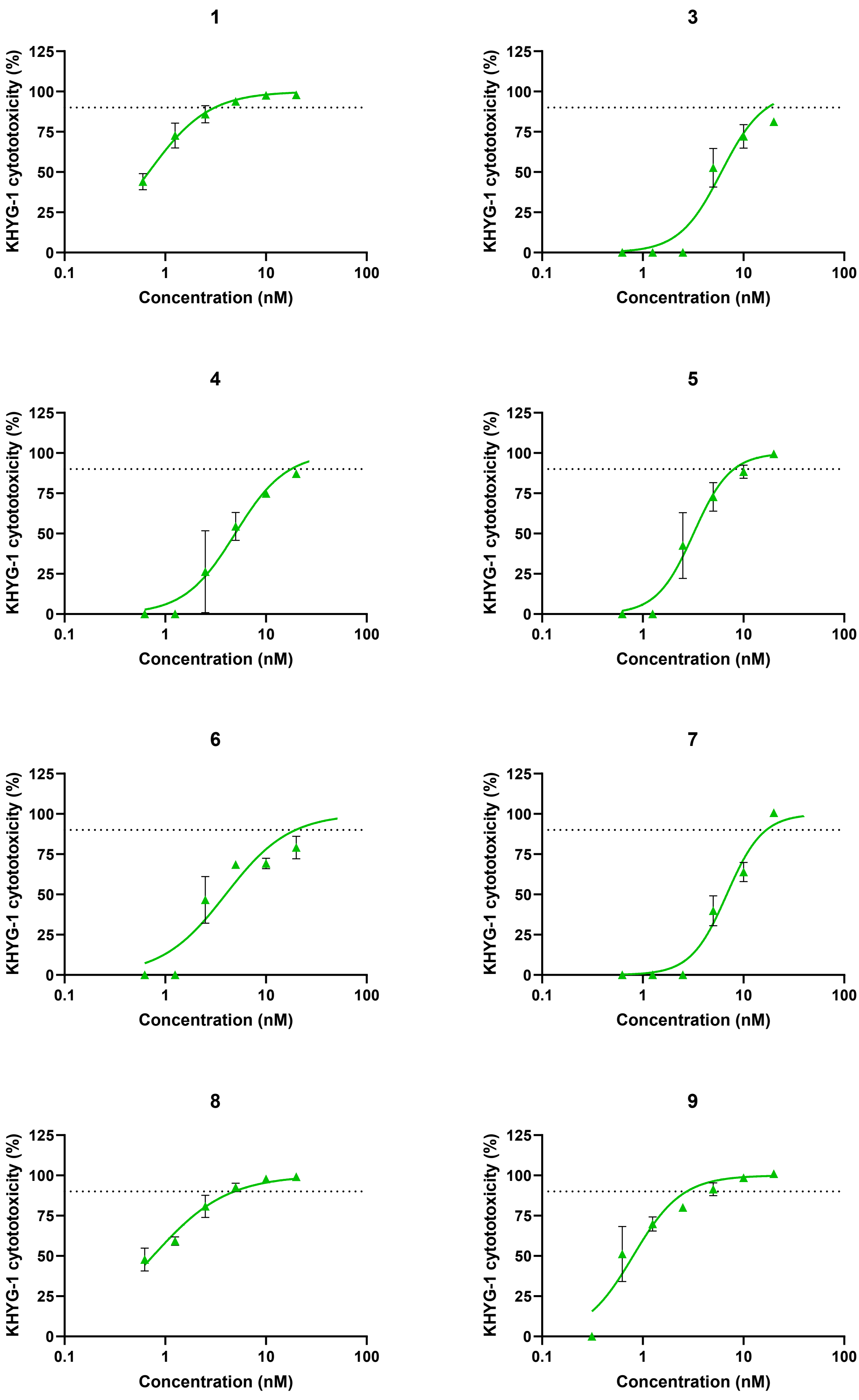

Figure S1: Representative KHYG-1 cytotoxicity plots for compounds **1**-**9**. The dotted line indicates inhibition of 90% of perforin-dependent cytotoxicity.

Table S1: *In vivo* perforin inhibition assay data and sample size for the 8 BZS compounds

**Compound**	**Dose**	**Schedule**	**Spleen**			**Blood**		
	(mg/kg)		**% Inhibition**	**± SEM**	**# Mice**	**% Inhibition**	**± SEM**	**# Mice**
1	160	qd x 4	48.20	3.46	39	55.47	6.58	28
	120	qd x 4	39.90	4.76	25	44.84	7.20	10
	80	qd x 4	7.66	7.03	32	26.33	14.43	10
3	120	qd x 4	0.53	3.47	60	-	-	-
	90	qd x 4	0.10	5.40	30	-	-	-
	60	qd x 4	0.07	8.82	30	-	-	-
4	100	qd x 4	80.53	4.51	12	-	-	-
5	100	qd x 4	14.88	11.59	9	-	-	-
6	100	qd x 4	22.70	18.37	5	-	-	-
7	160	qd x 4	26.55	11.34	15	-	-	-
8	160	qd x 4	39.14	15.23	5	-	-	-
9	120	qd x 4	61.28	24.39	5	-	-	-

Table S2: *In vivo* perforin inhibition assay data and sample size for the PK/PD relationship for compound **1**

**Compound**	**Dose**	**Schedule**	**Spleen**			**Blood**		
	(mg/kg)		**% Inhibition**	**± SEM**	**# Mice**	**% Inhibition**	**± SEM**	**# Mice**
1	160	bid	42.46	3.16	58	56.50	4.96	47
1	120	qd	16.71	10.39	19	17.84	12.56	19
	120	bid	30.50	3.74	57	43.66	7.65	37
	120	tid	40.42	6.57	21	69.14	8.47	21
1	80	qd	-4.19	10.55	12	-5.19	14.98	12
	80	bid	7.66	7.03	32	-0.71	15.73	17
	80	tid	-1.98	6.43	19	-1.34	13.27	19
1	120+80+80	tid	15.22	10.43	9	34.71	15.41	10
